# 3-Bromo­chroman-4-one

**DOI:** 10.1107/S1600536813005394

**Published:** 2013-03-02

**Authors:** Mahidansha M. Shaikh, Neil A. Koorbanally, Karen Du Toit, Deresh Ramjugernath, Johannes Bodenstein

**Affiliations:** a School of Chemistry and Physics, University of Kwazulu-Natal, Private Bag X54001, Durban 4000, South Africa; b Discipline of Pharmaceutical Science, University of KwaZulu-Natal, Private Bag X54001, Durban 4000, South Africa; c School of Engineering, University of KwaZulu-Natal, Durban 4041, South Africa

## Abstract

The heterocyclic ring of the title compound, C_9_H_7_BrO_2_, obtained by bromination of 4-chromanone with copper bromide, adopts a half-chair conformation. The supramol­ecular structure is governed by a weak C—H⋯O hydrogen bond. There is also π–π stacking between symmetry-related benzene rings; the centroid–centroid distance is 3.9464 (18), the perpendicular distance between the rings is 3.4703 (11) and the offset is 1.879 Å.

## Related literature

For similar structures, see: Schollmeyer *et al.* (2005 ▶); Piel *et al.* (2011 ▶); Betz *et al.* (2011 ▶). For synthesis involving chromanone inter­mediates, see: Simas *et al.* (2002 ▶); Zhang *et al.* (2008 ▶). For the biological activity of chromanone derivatives, see: Cho *et al.* (1996 ▶); Xu *et al.* (1998 ▶); Shaikh *et al.* (2012 ▶, 2013*a* ▶,*b* ▶).
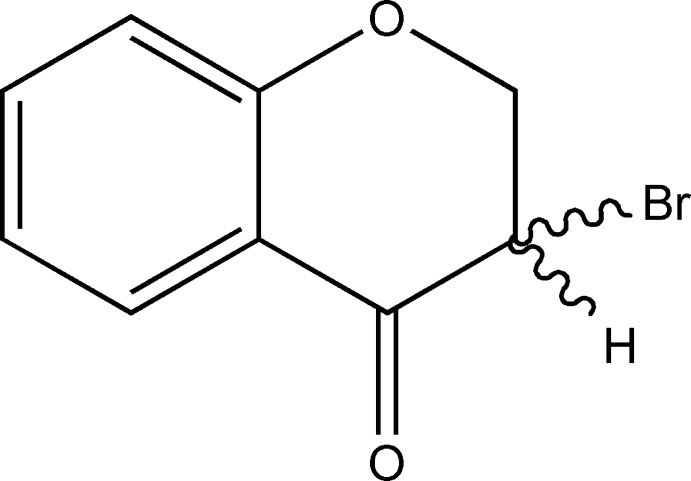


## Experimental

### 

#### Crystal data

C_9_H_7_BrO_2_
*M* *_r_* = 227.06
Monoclinic, 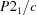

*a* = 10.0846 (7) Å
*b* = 7.9104 (6) Å
*c* = 10.9330 (8) Å
β = 110.164 (2)°
*V* = 818.71 (10) Å^3^
*Z* = 4
Mo *K*α radiation
μ = 4.97 mm^−1^
*T* = 173 K
0.16 × 0.12 × 0.12 mm

#### Data collection

Bruker Kappa DUO APEXII diffractometer
Absorption correction: multi-scan (*SADABS*; Sheldrick, 1997 ▶) *T*_min_ = 0.504, *T*_max_ = 0.587
5434 measured reflections
1659 independent reflections
1392 reflections with *I* > 2σ(*I*)
*R*_int_ = 0.026

#### Refinement

*R*[*F* ^2^ > 2σ(*F* ^2^)] = 0.025
*wR*(*F* ^2^) = 0.061
*S* = 1.05
1659 reflections
109 parameters
H-atom parameters constrained
Δρ_max_ = 0.39 e Å^−3^
Δρ_min_ = −0.39 e Å^−3^

### 

Data collection: *APEX2* (Bruker, 2006 ▶); cell refinement: *SAINT* (Bruker, 2006 ▶); data reduction: *SAINT*; program(s) used to solve structure: *SHELXS97* (Sheldrick, 2008 ▶); program(s) used to refine structure: *SHELXL97* (Sheldrick, 2008 ▶); molecular graphics: *ORTEP-3 for Windows* (Farrugia, 2012 ▶); software used to prepare material for publication: *SHELXL97*.

## Supplementary Material

Crystal structure: contains datablock(s) I, global. DOI: 10.1107/S1600536813005394/go2082sup1.cif

Structure factors: contains datablock(s) I. DOI: 10.1107/S1600536813005394/go2082Isup2.hkl

Supplementary material file. DOI: 10.1107/S1600536813005394/go2082Isup3.cml

Additional supplementary materials:  crystallographic information; 3D view; checkCIF report

## Figures and Tables

**Table 1:** Hydrogen-bond geometry (Å, °)

*D*—H⋯*A*	*D*—H	H⋯*A*	*D*⋯*A*	*D*—H⋯*A*
C2—H2*A*⋯O2^i^	0.99	2.44	3.311 (3)	146

Symmetry code: (i) 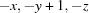.

**Fig. 1.**
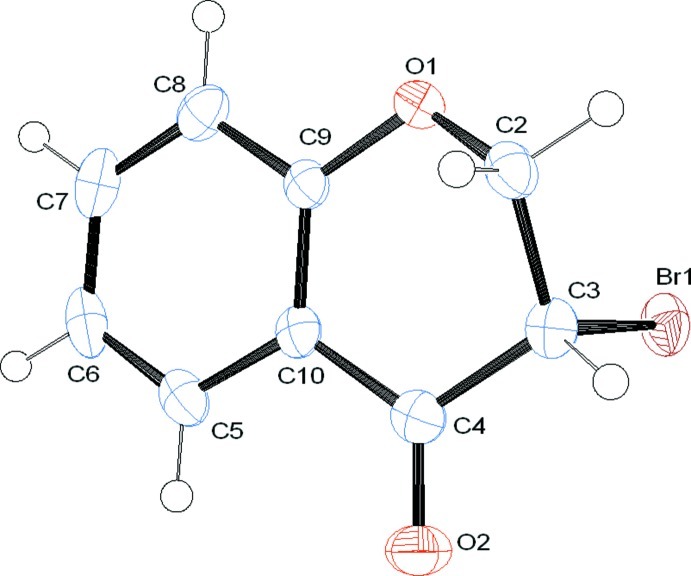
The molecular structure of the title compound, with atom labels and anisotropic displacement ellipsoids (drawn at 50% probability level).

## supplementary crystallographic information

### Comment

Many chromanone derivatives are used as versatile intermediates in the synthesis of natural products such as flavanone, isoflavanone and homoisoflavanones (Simas *et al.*, 2002, Zhang *et al.*, 2008). These derivatives possess anticancer and antibiotic properties (Cho *et al.*, 1996.). Chromanone derivatives also possess antiviral activities against HIV and the simian immunodeficiency virus (SIV) (Xu *et al.*, 1998). We recently reported the synthesis of several homoisoflavanone analogues from their corresponding chromanone derivatives with antiinflammatory (Shaikh *et al.*, 2012; Shaikh *et al.*, 2013*a*) and antifungal activities (Shaikh *et al.*, 2013*b*).

In the title compound, the pyranone moiety is fused with the benzene ring and adopts a half chair conformation. The dihedral angle between the benzene ring and the (C_3_—C_2_—O_1_) of the pyranone moiety is 43.03 (17)° and C2 flips out of the plane of the benzene ring by 0.5734 (31) Å (Fig. 1).

The supramolecular structure is governed by a weak C-H···O hydrogen bond, C2 –H2A···.O2 (-x,1-y,-z) with an H···O distance of 2.44 Å, a C···O distance of 3.311 (3)Å and an angle at H of 146°.

There is also π–π stacking between the two benzene rings across the centre-of-symmetry at (1/2,1/2,0), the centroid to centroid distance is 3.9464 (18)Å, the perpendicular distance between the rings is 3.4703 (11)Å and the offset is 1.879Å.

### Experimental

To a mixture of copper bromide (II) (11.351 g, 50.673 mmol) in ethyl acetate, chloroform (20:20 ml) was stirred under inert atmosphere at room temperature. Into this mixture, chroman-4-one (5 g, 33.783 mmol) in chloroform (20 ml) was added and the reaction mixture refluxed vigorously under inert atmosphere at 70 °C for 6 h. Completion of the reaction was monitored by thin layer chromatography. Upon completion, the reaction mixture was cooled, filtered and washed with chloroform (20 ml). The filtrate solution was evaporated under reduced pressure to get the pure title compound with a yield of 86%.

^1^H NMR (400 MHz, CDCl_3_): *δ* (p.p.m.): 4.53–4.65 (3*H*, m, H-2a, H-2 b & H-3), 6.98–7.06 (2*H*, m, H-6 & H-8), 7.48–7.52 (1*H*, m, H-7), 7.89 (1*H*, dd, *J* = 1.60, 7.92 Hz, H-5).

^13^C NMR (400 MHz, CDCl_3_): *δ* (p.p.m.): 45.43 (C-3), 71.26 (C-2), 117.95 (C-8), 11877 (C-10), 122.33 (C-6), 128.24 (C-7), 136.74 (C-5), 160.65 (C-9), 185.21 (C-4).

### Refinement

All non-hydrogen atoms were refined anisotropically. All hydrogen atoms were placed in idealized positions and refined with geometrical constraints. The structure was refined to a *R* factor of 0.0251.

### Crystal data

**Table:**

C_9_H_7_BrO_2_	*F*(000) = 448
*M**_r_* = 227.06	*D*_x_ = 1.842 Mg m^−^^3^
Monoclinic, *P*2_1_/*c*	Mo *K*α radiation, λ = 0.71073 Å
Hall symbol: -p 2ybc	Cell parameters from 5434 reflections
*a* = 10.0846 (7) Å	θ = 2.2–26.4°
*b* = 7.9104 (6) Å	µ = 4.97 mm^−^^1^
*c* = 10.9330 (8) Å	*T* = 173 K
β = 110.164 (2)°	Block, colourless
*V* = 818.71 (10) Å^3^	0.16 × 0.12 × 0.12 mm
*Z* = 4	

### Data collection

**Table:**

Bruker Kappa DUO APEXII diffractometer	1659 independent reflections
Radiation source: fine-focus sealed tube	1392 reflections with *I* > 2σ(*I*)
Graphite monochromator	*R*_int_ = 0.026
0.5° φ scans and ω scans	θ_max_ = 26.4°, θ_min_ = 2.2°
Absorption correction: multi-scan (*SADABS*; Sheldrick, 1997)	*h* = −12→8
*T*_min_ = 0.504, *T*_max_ = 0.587	*k* = −9→9
5434 measured reflections	*l* = −7→13

### Refinement

**Table:**

Refinement on *F*^2^	Primary atom site location: structure-invariant direct methods
Least-squares matrix: full	Secondary atom site location: difference Fourier map
*R*[*F*^2^ > 2σ(*F*^2^)] = 0.025	Hydrogen site location: inferred from neighbouring sites
*wR*(*F*^2^) = 0.061	H-atom parameters constrained
*S* = 1.05	*w* = 1/[σ^2^(*F*_o_^2^) + (0.0298*P*)^2^ + 0.3551*P*] where *P* = (*F*_o_^2^ + 2*F*_c_^2^)/3
1659 reflections	(Δ/σ)_max_ < 0.001
109 parameters	Δρ_max_ = 0.39 e Å^−^^3^
0 restraints	Δρ_min_ = −0.39 e Å^−^^3^

### Special details

**Table:**

Experimental. ^1^H NMR (400 MHz, CDCl3): δ (p.p.m.): 4.53–4.65 (3H, m, H-2a, H-2b & H-3), 6.98–7.06 (2H, m, H-6 & H-8), 7.48–7.52 (1H, m, H-7), 7.89 (1H, dd, J = 1.60, 7.92 Hz, H-5). 13C NMR (400 MHz, CDCl3): δ (p.p.m.): 45.43 (C-3), 71.26 (C-2), 117.95 (C-8), 118.77 (C-10), 122.33 (C-6), 128.24 (C-7), 136.74 (C-5), 160.65 (C-9), 185.21 (C-4).
Geometry. All e.s.d.'s (except the e.s.d. in the dihedral angle between two l.s. planes) are estimated using the full covariance matrix. The cell e.s.d.'s are taken into account individually in the estimation of e.s.d.'s in distances, angles and torsion angles; correlations between e.s.d.'s in cell parameters are only used when they are defined by crystal symmetry. An approximate (isotropic) treatment of cell e.s.d.'s is used for estimating e.s.d.'s involving l.s. planes.
Refinement. Refinement of *F*^2^ against ALL reflections. The weighted *R*-factor *wR* and goodness of fit *S* are based on *F*^2^, conventional *R*-factors *R* are based on *F*, with *F* set to zero for negative *F*^2^. The threshold expression of *F*^2^ > σ(*F*^2^) is used only for calculating *R*-factors(gt) *etc*. and is not relevant to the choice of reflections for refinement. *R*-factors based on *F*^2^ are statistically about twice as large as those based on *F*, and *R*- factors based on ALL data will be even larger.

### Fractional atomic coordinates and isotropic or equivalent isotropic displacement parameters (Å^2^)

**Table:**

	*x*	*y*	*z*	*U*_iso_*/*U*_eq_	
Br1	0.22559 (3)	0.04339 (3)	0.04673 (3)	0.03040 (11)	
O1	0.33588 (18)	0.3999 (2)	0.20473 (16)	0.0258 (4)	
O2	0.00306 (19)	0.3386 (2)	−0.13799 (18)	0.0348 (5)	
C2	0.2062 (3)	0.3261 (3)	0.2053 (2)	0.0261 (6)	
H2A	0.1470	0.4155	0.2238	0.031*	
H2B	0.2276	0.2418	0.2764	0.031*	
C3	0.1239 (3)	0.2415 (3)	0.0786 (2)	0.0251 (6)	
H3	0.0297	0.2053	0.0807	0.030*	
C4	0.1031 (3)	0.3566 (3)	−0.0374 (2)	0.0242 (5)	
C5	0.2106 (3)	0.5942 (3)	−0.1218 (3)	0.0274 (6)	
H5	0.1348	0.5872	−0.2026	0.033*	
C6	0.3170 (3)	0.7098 (3)	−0.1066 (3)	0.0330 (7)	
H6	0.3148	0.7825	−0.1764	0.040*	
C7	0.4279 (3)	0.7196 (3)	0.0121 (3)	0.0339 (7)	
H7	0.5018	0.7987	0.0223	0.041*	
C8	0.4323 (3)	0.6165 (3)	0.1148 (3)	0.0284 (6)	
H8	0.5080	0.6253	0.1955	0.034*	
C9	0.3249 (3)	0.4994 (3)	0.0995 (2)	0.0212 (5)	
C10	0.2132 (3)	0.4865 (3)	−0.0193 (2)	0.0210 (5)	

### Atomic displacement parameters (Å^2^)

**Table:**

	*U*^11^	*U*^22^	*U*^33^	*U*^12^	*U*^13^	*U*^23^
Br1	0.04127 (18)	0.01971 (15)	0.03327 (17)	0.00253 (11)	0.01674 (13)	0.00006 (12)
O1	0.0294 (10)	0.0252 (9)	0.0199 (9)	−0.0012 (8)	0.0046 (8)	0.0022 (8)
O2	0.0315 (11)	0.0358 (11)	0.0289 (11)	0.0004 (8)	−0.0002 (9)	−0.0033 (9)
C2	0.0339 (15)	0.0240 (13)	0.0218 (13)	0.0027 (11)	0.0116 (12)	0.0023 (11)
C3	0.0265 (13)	0.0225 (13)	0.0294 (14)	0.0016 (11)	0.0137 (12)	−0.0006 (11)
C4	0.0259 (13)	0.0233 (13)	0.0242 (13)	0.0058 (11)	0.0095 (12)	−0.0027 (11)
C5	0.0392 (15)	0.0233 (13)	0.0217 (13)	0.0094 (12)	0.0129 (12)	0.0008 (11)
C6	0.0521 (18)	0.0196 (13)	0.0364 (16)	0.0075 (12)	0.0269 (15)	0.0071 (12)
C7	0.0394 (16)	0.0199 (14)	0.0504 (18)	−0.0021 (12)	0.0255 (15)	−0.0019 (13)
C8	0.0276 (14)	0.0236 (13)	0.0348 (15)	−0.0006 (11)	0.0116 (12)	−0.0066 (12)
C9	0.0263 (13)	0.0175 (12)	0.0209 (12)	0.0033 (9)	0.0097 (11)	−0.0014 (9)
C10	0.0267 (13)	0.0172 (12)	0.0221 (13)	0.0032 (10)	0.0122 (11)	−0.0027 (10)

### Geometric parameters (Å, º)

**Table:**

Br1—C3	1.969 (2)	C5—C6	1.375 (4)
O1—C9	1.367 (3)	C5—C10	1.401 (4)
O1—C2	1.434 (3)	C5—H5	0.9500
O2—C4	1.218 (3)	C6—C7	1.393 (4)
C2—C3	1.505 (3)	C6—H6	0.9500
C2—H2A	0.9900	C7—C8	1.376 (4)
C2—H2B	0.9900	C7—H7	0.9500
C3—C4	1.515 (3)	C8—C9	1.390 (4)
C3—H3	1.0000	C8—H8	0.9500
C4—C10	1.476 (4)	C9—C10	1.399 (4)
			
C9—O1—C2	115.40 (19)	C6—C5—H5	119.7
O1—C2—C3	113.01 (19)	C10—C5—H5	119.7
O1—C2—H2A	109.0	C5—C6—C7	119.5 (2)
C3—C2—H2A	109.0	C5—C6—H6	120.2
O1—C2—H2B	109.0	C7—C6—H6	120.2
C3—C2—H2B	109.0	C8—C7—C6	121.1 (3)
H2A—C2—H2B	107.8	C8—C7—H7	119.5
C2—C3—C4	112.1 (2)	C6—C7—H7	119.5
C2—C3—Br1	111.18 (17)	C7—C8—C9	119.5 (3)
C4—C3—Br1	105.11 (15)	C7—C8—H8	120.3
C2—C3—H3	109.4	C9—C8—H8	120.3
C4—C3—H3	109.4	O1—C9—C8	116.7 (2)
Br1—C3—H3	109.4	O1—C9—C10	123.0 (2)
O2—C4—C10	123.6 (2)	C8—C9—C10	120.3 (2)
O2—C4—C3	121.3 (2)	C9—C10—C5	119.0 (2)
C10—C4—C3	115.2 (2)	C9—C10—C4	120.2 (2)
C6—C5—C10	120.6 (3)	C5—C10—C4	120.7 (2)
			
C9—O1—C2—C3	49.0 (3)	C7—C8—C9—O1	179.5 (2)
O1—C2—C3—C4	−51.4 (3)	C7—C8—C9—C10	−0.1 (4)
O1—C2—C3—Br1	66.0 (2)	O1—C9—C10—C5	179.9 (2)
C2—C3—C4—O2	−153.8 (2)	C8—C9—C10—C5	−0.5 (3)
Br1—C3—C4—O2	85.3 (2)	O1—C9—C10—C4	−2.5 (3)
C2—C3—C4—C10	27.4 (3)	C8—C9—C10—C4	177.1 (2)
Br1—C3—C4—C10	−93.5 (2)	C6—C5—C10—C9	0.6 (3)
C10—C5—C6—C7	0.0 (4)	C6—C5—C10—C4	−177.0 (2)
C5—C6—C7—C8	−0.6 (4)	O2—C4—C10—C9	179.9 (2)
C6—C7—C8—C9	0.7 (4)	C3—C4—C10—C9	−1.4 (3)
C2—O1—C9—C8	158.5 (2)	O2—C4—C10—C5	−2.5 (4)
C2—O1—C9—C10	−21.8 (3)	C3—C4—C10—C5	176.2 (2)

### Hydrogen-bond geometry (Å, º)

**Table:**

*D*—H···*A*	*D*—H	H···*A*	*D*···*A*	*D*—H···*A*
C2—H2*A*···O2^i^	0.99	2.44	3.311 (3)	146

Symmetry code: (i) −*x*, −*y*+1, −*z*.
